# Lower Serum Testosterone Associated with Elevated Polychlorinated Biphenyl Concentrations in Native American Men

**DOI:** 10.1289/ehp.0800134

**Published:** 2009-05-20

**Authors:** Alexey Goncharov, Robert Rej, Serban Negoita, Maria Schymura, Azara Santiago-Rivera, Gayle Morse, the Akwesasne Task Force on the Environment, David O. Carpenter

**Affiliations:** 1 Department of Environmental Health Sciences and; 2 Department of Biomedical Sciences, University at Albany, Rensselaer, New York, USA; 3 Wadsworth Center for Laboratories and Research, New York State Department of Health, Albany, New York, USA; 4 New York State Department of Health, Albany, New York, USA; 5 Department of Epidemiology and Biostatistics and; 6 Department of Education and Counseling Psychology, University at Albany, Albany, New York, USA; 7 Mohawk Nation at Akwesasne, Hogansburg, New York, USA; 8 Institute for Health and the Environment, University at Albany, Rensselaer, New York, USA

**Keywords:** DDE, endocrine disruption, hexachlorobenzene mirex, Native Americans

## Abstract

**Background:**

Polychlorinated biphenyls (PCBs) and chlorinated pesticides are endocrine disruptors, altering both thyroid and estrogen hormonal systems. Less is known of action on androgenic systems.

**Objective:**

We studied the relationship between serum concentrations of testosterone in relation to levels of PCBs and three chlorinated pesticides in an adult Native American (Mohawk) population.

**Methods:**

We collected fasting serum samples from 703 adult Mohawks (257 men and 436 women) and analyzed samples for 101 PCB congeners, hexachlorobenzene (HCB), dichlorodiphenyldichloroethylene (DDE), and mirex, as well as testosterone, cholesterol, and triglycerides. The associations between testosterone and tertiles of serum organochlorine levels (both wet weight and lipid adjusted) were assessed using a logistic regression model while controlling for age, body mass index (BMI), and other analytes, with the lowest tertile being considered the referent. Males and females were considered separately.

**Results:**

Testosterone concentrations in males were inversely correlated with total PCB concentration, whether using wet-weight or lipid-adjusted values. The odds ratio (OR) of having a testosterone concentration above the median was 0.17 [95% confidence interval (CI), 0.05–0.69] for total wet-weight PCBs (highest vs. lowest tertile) after adjustment for age, BMI, total serum lipids, and three pesticides. The OR for lipid-adjusted total PCB concentration was 0.23 (95% CI, 0.06–0.78) after adjustment for other analytes. Testosterone levels were significantly and inversely related to concentrations of PCBs 74, 99, 153, and 206, but not PCBs 52, 105, 118, 138, 170, 180, 201, or 203. Testosterone concentrations in females are much lower than in males, and not significantly related to serum PCBs. HCB, DDE, and mirex were not associated with testosterone concentration in either men or women.

**Conclusions:**

Elevation in serum PCB levels is associated with a lower concentration of serum testosterone in Native American men.

Testosterone, an androgenic steroid hormone, is secreted primarily by the testes in males and ovaries in females, although smaller amounts are produced by the adrenal glands in both sexes. Testosterone plays an important role in determination of secondary sex characteristics, in normal sexual functioning, and in overall physical and psychologic well-being. Testosterone is important throughout life. In prenatal and early and late postnatal periods, testosterone—and especially the ratio of testosterone to estrogen—determines sex differences and regulates growth of nearly all parenchymal organs and the skeletal muscle mass. In adults, testosterone regulates many functions, including libido, normal functioning of the immune system, level of aggression, and maintenance of muscle and bone mass.

Serum testosterone concentrations rise dramatically in puberty and then decrease somewhat with age (Alexandersen and Christiansen 2004; Allan and McLachlan 2004). On average, an adult human male produces about 8–10 times more testosterone than an adult female (Dabbs and Dabbs 2000). With sufficiently decreased concentrations of circulating testosterone in men, adverse health conditions result, including decreased physical endurance and memory capacity, loss of libido, depression of spermatogenesis following infertility (Small et al. 2007), loss of bone density (Morley et al. 1997; Schow et al. 1997), obesity (Kenny et al. 2001; Tsai et al. 2000), and depression (Barrett-Connor et al. 1999). Recently it has been reported that a decrease in testosterone is associated with an elevated risk for the development of Alzheimer’s disease (Moffat et al. 2004) and increased risk of heart attack (Hajjar et al. 1997; Simon et al. 1997; Zhao and Li 1998).

Polychlorinated biphenyls (PCBs) were manufactured in the United States from the late 1920s until the 1970s. During their period of use, large quantities of PCBs escaped into the environment. PCBs are persistent substances that bioaccumulate in both the environment and biota and they biomagnify in the food chain. Being lipophilic substances, they deposit in adipose tissue and in the lipid component of the serum. Because of their chemical structure, they are resistant to biodegradation and are therefore persistent in the human body (Johnson et al. 2006). Chlorinated pesticides were also widely used in the United States and other countries until the 1970s and, like PCBs, are persistent and widely distributed in human tissues (Sanz-Gallardo et al. 1999).

Akwesasne is a Native American (Mohawk) population of about 12,000 people residing along the St. Lawrence River in the Mohawk Territory at Akwesasne, near the junction of New York, Ontario, and Quebec. The territory is immediately downstream from three aluminum foundries, all of which used PCBs (Aroclor 1248) as hydraulic fluids, which leaked into the St. Lawrence River and its tributaries and have contaminated the local fish (Lacetti 1993). The Mohawk population is particularly vulnerable to PCB exposure because of their cultural and historical dependence on local fish, mammals, and waterfowl for food. Although their serum levels of PCBs are only moderately elevated [average, 5.29 ppb in males and 3.97 ppb in females (DeCaprio et al. 2005)], these values exceed the levels in persons without unusual exposure, which range from 0.9 ppb to 1.5 ppb (Agency for Toxic Substances and Disease Registry 2000). PCB levels in Mohawk breast milk (Hwang et al. 2001) and in serum (Fitzgerald et al. 2004) have been positively correlated with rates of consumption of local fish, although fish consumption has declined in recent years after consumption advisories were issued in the 1980s (Fitzgerald et al. 2004).

An inverse association between elevated concentrations of organochlorines and serum testosterone has been reported in animal studies. Kovacevic et al. (1995) demonstrated PCB inhibition of testosterone production by suspensions of Leydig cells from adult rat testis. In a follow-up study, Andric et al. (2000) showed that PCBs inhibit testosterone production by inhibitory effects on interstitial cell cultures, which resulted from an acute inhibition of enzymes responsible for the production of testosterone.

Compounds that bind to the aryl hydrocarbon receptor (AhR), such as 2,3,7,8-tetrachlorodibenzo-*p*-dioxin (TCDD) and dioxin-like coplanar PCBs, have anti-androgenic effects that are mediated through the AhR (Yeowell et al. 1987). Vincent et al. (1992) found an inverse association between serum PCB 77 (a coplanar congener) and circulating testosterone in exposed suckling rats. Hany et al. (1999) demonstrated that prenatal exposure of rats to Aroclor 1254 (40 mg/kg in food) caused decreased testosterone levels in adulthood. Murugesan et al. (2005) reported that 2 mg/kg Aroclor 1254 daily for 30 days resulted in lowered testosterone levels in adult male rats, whereas Ahmad et al. (2003) reported a similar finding in monkeys given Aroclor 1242 (200 μg/kg/day for 6 months).

Less information is available in humans. Richthoff et al. (2003) did not find a relation between serum PCB 153 (a non coplanar congener) and circulating testosterone in healthy young men, but did find a weak but significant negative correlation between PCB 153 levels and the testosterone to sex hormone–binding protein ratio. Dhooge et al. (2006) investigated reproductive function in young Flemish men in relation to measures of chemically activated luciferase expression (CALUX) dioxin toxic equivalents (TEQs) and found that a 2-fold increase in TEQs was associated with a 7.1% decrease in testosterone level.

For the pesticides, Asawasinsopon et al. (2006) did not find any relationship between levels of dichlorodiphenyldichloroethylene (DDE), the major metabolite of DDT (dichlorodiphenyltrichloethane), and testosterone. However DDE does exhibit potent androgen receptor antagonism (Kelce et al. 1995). Hexachlorobenzene (HCB) has been reported to have mixed agonist/antagonist androgenic actions (Ralph et al. 2003), but there is little information available on effects of mirex on androgenic systems.

Elevated PCB levels have been correlated with reduced sperm mobility (Bush et al. 1986; Richthoff et al. 2003). In a study from Taiwan, Hsu et al. (2003) observed that men exposed to PCBs and furans in 1978–1979 had more abnormal sperm in 1999–2000 than nonexposed men. Their sperm showed a reduced capability to bind and penetrate oocytes. This may be an indirect indication of reduced serum testosterone.

In the present study we examined whether there is a relationship between testosterone concentrations and serum levels of total PCBs, 12 single PCB congeners, 5 PCB groups, and 3 pesticides (HCB, DDE, and mirex) in an adult population of male and female Mohawks.

## Materials and Methods

The study subjects were Mohawk adults 18–95 years of age who resided at Akwesasne for at least 5 years. There were 257 males, ages 18–85 years, and 436 females, ages 18–95 years, for a total of 703 individuals enrolled in the study. Males and females were analyzed separately because they have very different testosterone levels, different physiologic and biochemical mechanisms of sex hormone regula tion, different medication approaches, and different organochlorine dynamics. Subject recruitment took place between 1995 and 2000, and sampling was performed by random selection of households as previously described (Codru et al. 2007; DeCaprio et al. 2005; Goncharov et al. 2008). Informed consent was obtained in writing from all participants.

Fasting blood samples were obtained by venipuncture between 0700 and 1030 hours. Blood was drawn in 5-mL collection tubes for analysis of serum testosterone, cholesterol, and triglycerides. In addition, a 10-mL sample (for ~ 5 mL of serum) was drawn for analysis of 101 PCB congeners, HCB, DDE, and mirex. The blood samples were allowed to clot at room temperature for 1 hr, then centrifuged, and the serum was removed. For both analyses, serum was stored at −80°C on-site until transported on dry ice to the laboratories for analysis.

We measured total testosterone in unextracted serum specimens using a solid-phase radio immunoassay procedure with a specific rabbit antibody affixed to polypropylene tubes (Siemens Diagnostics/Diagnostic Products, Los Angeles, CA, USA). I^125^-labeled testosterone was used as a tracer, and radioactivity due to bound tracer was measured using a Wallace 1470 Wizard gamma counter (Wallace/Perkin-Elmer, Waltham, MA). Logit-log transformations, standard curves, and results were calculated by instrument-based software. All measurements were made in duplicate and the average result reported. If the duplicate measurement values differed by > 25% (or by 25 ng/dL at concentrations < 100 ng/dL), that analysis was not accepted and specimens were re-assayed. The variation on duplicate samples was 4.8% on samples with > 100 ng/dL, and 7.7% for those < 100 ng/dL. The limit of quantitation (functional sensitivity) was 10 ng/dL for testosterone; for statistical purposes, results < 10 ng/dL were set at one half of the limit of quantitation, that being 5 ng/dL.

We measured concentrations of cholesterol and triglycerides using a Hitachi 911 analyzer (Roche Diagnostics, Indianapolis, IN). Cholesterol was determined with cholesterol esterase and cholesterol oxidase coupled with peroxidase (Allain et al. 1974). Triglycerides were determined by a glycerol kinase-based procedure that corrects for the free glycerol in the specimen (Kohlmeier 1986), as recommended by the National Cholesterol Education Program Working Group on Lipoprotein Measurement (Stein and Myers 1996). The facility, located in the Wadsworth Laboratories of the New York State Department of Health, is approved by the Clinical Laboratory Improvement Amendments and is a member of the Centers for Disease Control and Prevention reference laboratory network for lipid measurements (Myers et al. 2000). Total serum lipids were calculated using the formula proposed by Philips et al. (1989) and recently validated by the same group (Bernert et al. 2007).

PCB analysis was performed in the Exposure Assessment Laboratory of the University at Albany, Rensselaer, New York. Concentrations of serum PCB congeners were determined using ultratrace analytical methods using dual-column gas chromatography with electron-capture detection. The use of dual columns allows definitive determination of 91 analytical peaks that measure levels of 101 PCB congeners, DDE (the major present metabolite of DDT), mirex, and HCB (DeCaprio et al. 2000). Of these, 83 peaks represent single PCB congeners, whereas 18 peaks reflect the presence of 2 or 3 different congeners. The limit of detection (LOD) for individual congeners ranged from 0.01 ppb to 0.15 ppb (median, 0.02 ppb). The results are reported both on the basis of wet weight, treating total serum lipids as a covariate, and after lipid adjustment. The toxicants studied are fat-soluble substances found in the lipid layer, and theoretically the measurement should be more accurate if PCBs are adjusted for the amount of lipids in the serum sample. Recent reports, however, have suggested that lipid-adjusted levels are particularly prone to bias (Schisterman et al. 2005), so we reported both wet-weight values with lipids as a covariate and lipid-adjusted values. Lipid-based values were determined by dividing the wet-weight value of PCBs by total serum lipids as calculated from the above-mentioned Philips formula, then multiplying by a factor of 10^5^ for unit adjustment (nanograms of toxicant per gram of lipid). Values < LOD were set at LOD/2.

We evaluated the relationship between serum testosterone and PCB concentration by consideration of total PCBs (the sum of the 101 congeners for which we had analyses). In addition, we considered separately the concentrations of 12 individual congeners: PCBs 52 and 105 (lower-chlorinated and less-persistent congeners), PCBs 74, 99, and 118 [they have been reported to best correlate with rates of local fish consumption (Fitzgerald et al. 2006)], PCBs 138, 153, and180 [these are the congeners present at the highest concentration in Mohawks (DeCaprio et al. 2005)], and PCBs 170, 201, 203, and 206 [these are highly chlorinated, persistent, and present in most of the population (DeCaprio et al. 2005)]. Then we analyzed the relationships for several groups of congeners that are believed to have either common modes of action or structural similarity. These included non persistent potentially estrogenic congeners (NpPEC; the sum of concentrations of five congeners, PCBs 31, 44, 49, 52, and 70), a category first described by Wolff et al. (1997) and one that has been studied in relation to thyroid disease in the Mohawk population by Negoita (2007). In addition we distinguished mono-*ortho*-substituted congeners (the sum of 22 congeners, PCBs 1, 6, 7, 8, 9, 22, 25, 26, 28, 29, 31, 33, 56, 63, 66, 67, 70, 74, 105, 114, 118, and 156); di-*ortho* congeners (the sum of 43 congeners, PCBs 4/2, 10, 17, 18, 19, 24/27, 32/16, 40, 42, 44, 47/59, 49, 52, 64, 71, 83, 87, 90/101, 92, 97, 99, 110, 128, 129, 130, 137, 163/164/138, 141, 146, 153, 158, 170, 172, 180, 190, and 194); and tri- and tetra-*ortho* congeners (the sum of 27 congeners, PCBs 45, 46, 51, 53, 84, 91, 95, 132, 134, 136, 144, 151, 171, 174, 176, 177, 179, 183, 185, 187, 195, 196, 199, 200, 201, 203, and 206). We also evaluated dioxin TEQs (Van den Berg et al. 2006) [the sum of concentrations of five congeners for which TEQs are assigned, multiplied by their TEQs: PCBs 77 (0.0001), 105 (0.00003), 114 (0.00003), 118 (0.00003), and 156 (0.00003)]. These PCB congener groupings have been used in our previous studies of the Mohawk population (Codru et al. 2007; Goncharov et al. 2008; Negoita, 2007). We considered each of the three pesticides separately.

Because the testosterone distribution was skewed in both men and women and because the Shapiro-Wilk test was < 0.05 even after various transformations, testosterone was dichotomized at the median into low and high. Because age and body mass index (BMI) influence testosterone concentrations as well as toxicant levels, they are potential confounders and were included as covariates in the models. Both age and BMI were treated as continuous variables.

### Statistical analysis

We performed bivariate analyses for total PCBs, 12 individual congeners, five PCB congener groups, and three pesticides. The toxicants were grouped in tertiles of exposure, with the lowest serving as the reference (comparison) group. We used PROCGENMOD (SAS Institute Inc., Cary, NC) to run logistic regression and log-binomial analysis to obtain odds ratios (ORs) and relative risks (RRs), respectively. We estimated the association of testosterone with serum wet-weight concentrations of total PCBs, PCBs 52, 74, 99, 105, 118, 138, 153, 170, 180, 201, 203, and 206, NpPEC, mono-*ortho*-substituted congeners, di-*ortho*-substituted congeners, tri- and tetra-*ortho*-substituted congeners, dioxin TEQs, DDE, HCB, and mirex, while adjusting for age, BMI, and total lipids. We also adjusted for the other toxicants (total PCBs, HCB, DDE, and mirex) when determining ORs for each; for the single congeners and congener group, we adjusted for HCB, DDE, and mirex. We ran two separate models for males and females. Finally, we replicated the analysis using lipid-standardized concentration values for the toxicants. All analyses were conducted in SAS (version 9.1, SAS Institute Inc.).

## Results

**Figure 1:**
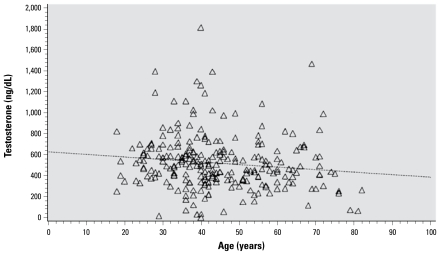
Serum testosterone concentration as a function of age in Mohawk men.

Table 1 shows the characteristics of subjects (age, BMI, testosterone, and total lipid), separated by sex. Reference normal values for testosterone are reported to be 260–1,600 ng/dL for males and 20–80 ng/dL for females (Burtis and Ashwood 1999). Because we detected no statistically significant relationship between toxicants and testosterone levels in females, no further data on females will be presented. Figure 1 shows the age dependence of testosterone levels in men in our population. Although there is some reduction in testosterone levels with age, it is modest. Table 2 shows the distribution of total PCBs, the 12 single congeners, various PCB groups, and three pesticides in males, as well as the percentage of samples whose value was > LOD. Mean values are presented with ranges for both wet-weight and lipid-adjusted concentrations.

**Figure 2:**
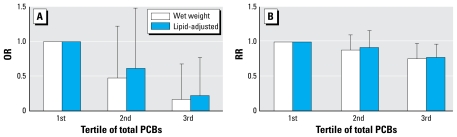
ORs (*A*) and RRs (*B*) showing association of testosterone levels in men with tertile of total PCBs expressed as either wet-weight (with lipids as a covariate) or lipid-adjusted values. Error bars show 95% CIs.

Figure 2 shows the association between testosterone concentration and levels of total serum PCBs in tertiles in males. Figure 2A shows results by logistic regression, whereas Figure 2B shows the more stringent log-binomial regression. We observed a significant inverse association (OR = 0.17; RR = 0.77) between serum testosterone and highest versus lowest tertile of total wet-weight PCBs after adjustment for age, BMI, total serum lipids, and concentrations of the three pesticides. When the PCB concentration was expressed after lipid adjustment, the relationship was very similar (OR = 0.22; RR = 0.80) after concurrent adjustment for pesticides. There was no significant association between testosterone and any of the three pesticides, whether considered as wet-weight or lipid-adjusted concentrations. For wet-weight measurements for HCB, OR = 0.54 [95% confidence interval (CI), 0.19–1.55]; for DDE, OR = 1.18 (95% CI, 0.37–3.69); and for mirex, OR = 1.30 (95% CI, 0.48–3.48) comparing highest with lowest tertiles.

Of the 12 single congeners and five congener groups studied, we found no significant relationship with serum testosterone levels for PCBs 52, 105, 118, 138, 170, 180, 201, and 203 or the NpPEC group (data not shown). Using logistic regression, we found significant associations between testosterone and four individual congeners (PCBs 74, 99, 153, and 206) and four congener groups (mono-*ortho*-substituted, di-*ortho*-substituted, tri- and tetra-*ortho*-substituted, and dioxin-like TEQs) (Table 3). Using log-binomial analysis, we found significant RRs for PCBs 153 and 206 and the tri- plus tetra- congener group. The other significant relations observed with logistic regression showed nonsignificant reductions with log-binomial analysis. To assess the presence of possible multicollinearity, we determined the variance inflation factor (VIF). We found no multicollinearity, and the VIF did not exceed 5 in any of our models.

A critical factor in understanding which of the PCB congeners and congener groups is responsible for the relationships observed is the degree to which the concentrations of one compound correlate with those of the others. Table 4 shows the correlation coefficients for all of the groups we have studied.

## Discussion

Total serum PCBs, four individual PCB congeners, and several PCB groups were negatively correlated with serum testosterone concentrations in adult males after adjustment for age, BMI, and concentrations of three pesticides. This was true whether we used wet-weight concentrations with total lipids as a covariate or lipid-adjusted values, and we found significant relations whether we used logistic regression or log-binomial analysis. Of the three pesticides studied, none showed a significant relation ship with testosterone concentration. No statistically significant relations between PCBs and testosterone were detected in women, probably because their testosterone levels are much lower than those in men and because testosterone levels vary with the menstrual cycle.

Endocrine disruptors are compounds that are able to mimic or block these natural biological processes. They may act through agonist or antagonist action at receptors or by altering rates of synthesis or metabolism of the steroid hormone (Nesaretnam et al. 1996; Roy et al. 2004). They may also compete for serum hormone-binding proteins or induce metabolic pathways that degrade the endogenous hormone (Duan et al. 1999; Kester et al. 2000; Masuyama et al. 2000). Most sex hormone–endocrine-disruptor studies have focused on estrogens. However, because sexual development in both males and females is dependent on the ratio of estrogens to androgens, it is also important to understand how organochlorines such as PCBs and organochlorine pesticides influence concentrations of androgens.

At high doses, dioxin and dioxin-like PCBs are known to decrease circulating androgen concentrations in rats due to a decrease in the function of steroidogenic enzymes in Leydig cells (Moore et al. 1985). Roman and Peterson (1998) demonstrated that dioxin (64 ng/kg) given only once to pregnant rat dams produced anti androgenic effects in male offspring. These adverse effects include decreased weights in accessory sex organs, delayed preputial separation, decreased testicular sperm production, and decreased epididymal sperm storage. *Ortho*-substituted PCB congeners, which have weak or no affinity for the AhR, can affect germinal cells indirectly through formation of reactive oxygen species (Fischer et al. 1998) or directly by altering cell lipid membranes (Tan et al. 2004). Kuriyama and Chahoud (2004) reported that male rats exposed to PCB 118 *in utero* showed smaller testes and epididymides, larger seminal vesicles, decreased sperm and spermatid numbers, and impaired daily sperm production as adults.

The only other human study of PCBs and testosterone, to our knowledge, is that of Richthoff et al. (2003). They reported depression of the ratio of serum testosterone to sex hormone–binding globulin in Swedish men, 18–21 years of age, in relation to levels of PCB 153, but did not detect any relationship to total testosterone level. Dhooge et al. (2006) reported reduced testosterone in relation to CALUX-TEQ values, but these results were primarily due to dioxins. In other human studies of male reproductive function, Yu-Cheng boys exposed *in utero* to PCBs and furans showed reduced penile length at 11–14 years of age (Guo et al. 2000). It is interesting that Mocarelli et al. (2008) did not find a relationship between serum dioxin and testosterone levels in exposed residents in Seveso, Italy, in spite of effects on sperm concentration and motility.

We found significant relationships between serum testosterone concentrations in men with four non–dioxin-like PCB congeners and for all PCB groups except NpPEC. PCBs 74 and 99, mono- and di-*ortho*, respectively, are the congeners most closely associated with levels of fish consumption in this population (Fitzgerald et al. 2004). PCB 153, a very persistent di-*ortho* congener, is present in the Mohawks at a higher concentration than any other congener. PCB 206 is a very highly chlorinated congener with three *ortho* chlorines and was present in a rather low concentration, yet had a strong inverse relation with serum testosterone. The strong associations for the di-*ortho* and tri- plus tetra-*ortho* congener groups also suggest that the relationship is not dependent primarily or only on AhR activity.

The information presented in Table 4, which shows the correlations among concentrations of the various congeners and groups, provides additional support for our conclusion that the inverse relationship between total PCBs and testosterone level is not dependent only on dioxin-like activity. Although PCB 153 is rather highly correlated with total PCBs and shows a clear inverse relation to serum testosterone, PCBs 138 and 180, both present at high concentrations, were even more highly correlated with the total PCB concentration. However, neither was significantly correlated with testosterone concentration. PCB 206 was one of the congeners least tightly correlated with total PCBs, yet was strongly inversely correlated with testosterone levels.

These lipophilic substances, including the three pesticides, migrate together, and this makes it difficult to distinguish actions of single congeners and/or individual pesticides. However, our observations provide support for the view that one can obtain important information on actions of individual congeners or groups of congeners through careful analysis. The results reported here show that the different congeners have independent actions, even though currently we do not understand all of the mechanisms involved.

The physiologic significance of the relationships we observed is not clear. The mean testosterone levels between the highest and lowest PCB tertiles differ by about 25%, but testosterone levels in the general population do vary widely, and it is not certain what effects would result from reductions in testosterone levels of this magnitude. However, our results demonstrate that PCBs disrupt androgenic systems, not just estrogen and thyroid hormonal systems.

None of the chlorinated pesticides studied had any effect on serum testosterone, in spite of the fact that they are lipophilic substances that co-migrate with PCBs. As shown in Table 4, the pesticide levels are only partially correlated with levels of PCBs, probably reflecting the fact that the aluminum companies at Akwesasne constitute an exceptional source of PCB exposure while the pesticide exposure there is not unusually elevated. Nonetheless, it is clear that residents of Akwesasne, as elsewhere, are exposed to mixtures of chemicals from fish, other foods, and air, and that the consequent health outcomes may reflect from exposure to more than one chemical contaminant and interactions among the chemicals.

There are major strengths to our study. The sample size was large enough to allow us to separate males and females and still have adequate power. The organochlorine determinations were performed at one time point using the same analytical methods, which minimizes the potential for measurement bias. We measured 101 PCB congeners and three pesticides. The relationships observed were similar, whether contaminant levels were expressed on a wet-weight or lipid-adjusted basis, and demonstrated reasonable consistency. The similarity of results obtained through these two methods of data presentation adds to the confidence that the conclusions are real.

The present study also has several limitations. Only single measurements were made for all biochemical analytes and for serum PCBs and pesticides. Participants were instructed to fast overnight before providing blood for analy sis, but we could not confirm that this was always the case. If some of them did not fast as instructed, measurement bias could affect our findings. However, this bias is likely to be nondifferential because it is unlikely that participants with higher toxicant levels would preferentially fail to follow fasting instructions. Our method of measurement of serum PCBs provides information on 101 PCB congeners, but it does not include some potent dioxin-like congeners (e.g., PCB 126). Thus, we do not have full information on dioxin-like activity. In addition, we did not have direct measurement of total serum lipids, but used the formula developed by Phillips et al. (1989) to determine total serum lipids.

Despite the limitations, the general pattern of results between organochlorine and testosterone concentrations in men is significant and consistent. The analysis of the results with single PCB congeners and groups of congeners shows that the relationship is not due solely to dioxin-like PCBs.

## Conclusion

In this cross-sectional study, we found serum concentrations of total PCBs, four single PCB congeners, and four PCB congener groups to be negatively associated with testosterone concentration in adult male Native Americans. Concentrations of eight other PCB congeners, one congener group, and three pesticides were not correlated with testosterone concentration. These findings indicate that exposure to some, but not all, PCB congeners is associated with a lower concentration of circulating testosterone in males.

## Figures and Tables

**Table 1:** Selected characteristics of study participants (703 adult Mohawks).

Characteristic^a^	Median	Mean ± SE	Range
Males (*n* = 257)			
Age (years)	42	44.3 ± 0.9	18–82
BMI (kg/m^2^)	30	30.9 ± 0.4	15.6–54.8
Testosterone (ng/dL)	494	524 ± 16.6	5–1817
Lipids (mg/L)^b^	600.6	608.8 ± 9.8	310.9–1130.9
Females (*n* = 446)			
Age (years)	42	44.5 ± 0.7	18–95
BMI (kg/m^2^)	29	29.9 ± 0.3	14.6–59.8
Testosterone (ng/dL)	26	34.7 ± 3.0	5–824
Lipids (mg/L)^b^	565.1	587.8 ± 7.7	269.9–1378.3

a Sample size < 703 for some characteristics because of missing data.

b Estimated total lipids based on direct measurement of serum total cholesterol and triglycerides.

**Table 2:** Distribution of serum wet-weight (ppb) and lipid-adjusted (ng/g lipids) concentrations of PCBs and chlorinated pesticides in Mohawk men.

Analyte	Percent > LOD	Wet weight [mean (range)]	Lipid adjusted [mean (range)]
PCBs			
Total PCBs	100	5.93 (1.53–48.87)	953.18 (216.58–7907.94)
PCB 52	72.76	0.04 (0.01–0.24)	6.65 (0.94–40.22)
PCB 74	97.28	0.33 (0.01–4.79)	51.65 (1.88–775.18)
PCB 99	96.88	0.28 (0.01–2.58)	43.49 (1.34–417.53)
PCB 105	86.77	0.08 (0.01–1.55)	12.11 (1.31–0.84)
PCB 118	100	0.30 (0.03–5.16)	47.41 (4.64–835.06)
PCB 138	100	0.65 (0.08–5.92)	102.19 (11.85–958.05)
PCB 153	99.61	0.78 (0.01–6.68)	123.69 (1.19–1081.04)
PCB 170	98.05	0.16 (0.01–1.44)	25.35 (1.19–233.04)
PCB 180	100	0.61 (0.03–4.48)	96.03 (4.57–725.01)
PCB 201	73.54	0.05 (0.01–0.52)	7.81 (1.09–67.97)
PCB 203	84.05	0.08 (0.01–0.98)	13.04 (1.09–151.93)
PCB 206	70.82	0.07 (0.01–1.15)	11.42 (1.14–191.84)
NpPEC	81.71	0.13 (0.06–0.76)	108.91 (45.01–600.16)
Mono-o*rtho*	100	1.24 (0.32–14.56)	200.36 (50.22–2356.28)
Di-*ortho*	100	3.46 (0.77–25.23)	554.05 (110.03–4082.22)
Tri- and tetra-*ortho*	100	1.11 (0.28–8.56)	178.93 (40.82–1384.48)
Dioxin-like (TEQ)	100	1.9 × 10^−5^ (3.4 × 10^−6^–2.5 × 10^−4^)	3 × 10^−3^ (5.1 × 10^−4^–4.1 × 10^−2^)
Chlorinated pesticides			
HCB	98.44	0.08 (0.01–0.28)	12.4 (1.38–71.63)
DDE	100	2.89 (0.14–14.98)	466.24 (22.31–2322.41)
Mirex	86.62	0.18 (0.01–1.67)	28.43 (1.09–270.26)

**Table 3:** Levels of serum testosterone in relation to serum concentrations of PCBs in Mohawk men.

Analyte	Median (ng/dL)	Wet-weight measurement^a^ [OR (95% Cl)]	Lipid-adjusted measurement^b^ [OR (95% Cl)]
PCB 74			
Lowest tertile	574	1.00	1.00
Medium tertile	474	0.51 (0.21–1.20)	0.52 (0.22–1.19)
Highest tertile	406	0.28 (0.08–0.90)*	0.29 (0.09–0.93)*
PCB 99			
Lowest tertile	583	1.00	1.00
Medium tertile	486	0.50 (0.22–1.21)	0.64 (0.30–1.36)
Highest tertile	421	0.33 (0.11–1.00)*	0.41 (0.14–1.07)
PCB 153			
Lowest tertile	551	1.00	1.00
Medium tertile	499	0.48 (0.17–1.35)	0.52 (0.19–1.39)
Highest tertile	431	0.15 (0.03–0.69)*	0.19 (0.05–0.77)*
PCB 206			
Lowest tertile	543	1.00	1.00
Medium tertile	517	0.63 (0.26–1.53)	0.58 (0.22–1.33)
Highest tertile	438	0.20 (0.06–0.67)*	0.27 (0.08–0.83)*
Mono-*ortho*			
Lowest tertile	551	1.00	1.00
Medium tertile	509	0.83 (0.38–1.82)	0.79 (0.23–1.39)
Highest tertile	396	0.35 (0.12–0.97)*	0.37 (0.13–1.00)*
Di-*ortho*			
Lowest tertile	574	1.00	1.00
Medium tertile	499	0.57 (0.23–1.39)	0.69 (1.28–1.67)
Highest tertile	432	0.30 (0.08–0.91)*	0.23 (0.06–0.80)*
Tri-tetra-*ortho*			
Lowest tertile	551	1.00	1.00
Medium tertile	501	0.36 (0.26–1.24)	0.48 (0.20–1.17)
Highest tertile	434	0.30 (0.08–0.97)*	0.20 (0.06–0.71)*
Dioxin-like (TEQ)			
Lowest tertile	561	1.00	1.00
Medium tertile	474	0.68 (0.26–1.29)	0.44 (0.20–0.95)*
Highest tertile	413	0.32 (0.11–0.90)*	0.10 (0.16–0.90)*

a All ORs were adjusted for age, BMI, total serum lipids, and concentrations of HCB, DDE, and mirex.

b Toxicant concentrations in serum lipids. All ORs were adjusted for age and BMI, as well as concentrations of HCB, DDE, and mirex.

* Statistically significant (*p* < 0.05).

**Table 4:** Correlation coefficients of PCBs and chlorinated pesticides.

Analyte	TPCB	52	74	99	105	118	138	153	170	180	201	203	206	HCB	DDE	Mirex
TPCB	–	0.13	0.89	0.92	0.84	0.89	0.98	0.97	0.95	0.93	0.82	0.74	0.61	0.66	0.68	0.81
52	0.13	–	0.04	0.07	0.12	0.13	0.02	0.03	0.01	–0.01	0.02	–0.01	–0.02	0.06	0.002	0.06
74	0.89	0.04	–	0.83	0.85	0.89	0.87	0.84	0.81	0.77	0.74	0.57	0.46	0.64	0.60	0.65
99	0.92	0.07	0.83	–	0.82	0.88	0.94	0.91	0.83	0.81	0.74	0.59	0.45	0.68	0.71	0.75
105	0.84	0.12	0.85	0.82	–	0.97	0.81	0.78	0.73	0.66	0.65	0.51	0.38	0.53	0.55	0.68
118	0.89	0.13	0.89	0.88	0.97	–	0.87	0.84	0.79	0.73	0.73	0.56	0.42	0.60	0.62	0.72
138	0.98	0.02	0.87	0.94	0.81	0.87	–	0.98	0.94	0.93	0.82	0.69	0.55	0.67	0.71	0.80
153	0.97	0.03	0.84	0.91	0.78	0.84	0.98	–	0.96	0.95	0.81	0.71	0.57	0.66	0.70	0.80
170	0.95	0.01	0.81	0.83	0.73	0.79	0.94	0.96	–	0.96	0.79	0.75	0.60	0.60	0.63	0.77
180	0.98	–0.01	0.77	0.81	0.66	0.73	0.93	0.95	0.96	–	0.76	0.79	0.67	0.60	0.63	0.77
201	0.82	0.02	0.74	0.74	0.65	0.73	0.82	0.81	0.79	0.76	–	0.59	0.52	0.54	0.55	0.70
203	0.74	–0.01	0.57	0.59	0.51	0.56	0.69	0.71	0.75	0.79	0.59	–	0.85	0.45	0.48	0.55
206	0.61	–0.02	0.46	0.45	0.38	0.42	0.55	0.57	0.60	0.67	0.52	0.85	–	0.37	0.40	0.44
HCB	0.66	0.06	0.64	0.68	0.53	0.60	0.67	0.66	0.60	0.60	0.54	0.45	0.37	–	0.71	0.48
DDE	0.68	0.002	0.60	0.71	0.55	0.62	0.71	0.70	0.63	0.63	0.55	0.4	80.40	0.71	–	0.49
Mirex	0.81	0.06	0.65	0.74	0.68	0.72	0.80	0.80	0.77	0.77	0.70	0.55	0.44	0.48	0.49	–

TPCB, total serum PCB concentration.
